# The Localization of Cell Wall Components in the Whole-Mount Immunolabeled *Nepenthes* Digestive Glands

**DOI:** 10.3390/ijms26189174

**Published:** 2025-09-19

**Authors:** Bartosz J. Płachno, Małgorzata Kapusta, Marcin Feldo, Piotr Stolarczyk, Piotr Świątek

**Affiliations:** 1 Department of Plant Cytology and Embryology, Institute of Botany, Faculty of Biology, Jagiellonian University, 9 Gronostajowa St., 30-387 Kraków, Poland; 2 Bioimaging Laboratory, Faculty of Biology, University of Gdańsk, 59 Wita Stwosza St., 80-308 Gdańsk, Poland; 3 Department of Vascular Surgery and Angiology, Medical University of Lublin, 16 Staszica St., 20-081 Lublin, Poland; 4 Department of Botany, Physiology and Plant Protection, Faculty of Biotechnology and Horticulture, University of Agriculture in Kraków, 29 Listopada 54 Ave., 31-425 Kraków, Poland; 5 Institute of Biology, Biotechnology and Environmental Protection, Faculty of Natural Sciences, University of Silesia in Katowice, 9 Bankowa St., 40-007 Katowice, Poland

**Keywords:** pitcher plants, carnivorous plants, cell wall, cell wall microdomains, digestive glands, hemicelluloses, scanning transmission electron microscopy, pectic homogalacturonan, xyloglucan, xylan

## Abstract

Glands from *Nepenthes* pitcher secrete various substances, including digestive enzymes, and absorb nutrients from digested prey. Due to the extreme specialization of these glands, they are an interesting model for studying secretory cells’ structure and activity. This study aimed to fill the gap in the literature concerning the immunocytochemistry of *Nepenthes* digestive glands in the major cell wall polysaccharides and glycoproteins. To do this, the localization of the cell wall components in the cell walls of glandular cells was performed using whole-mount immunolabeled glands of *Nepenthes albomarginata*. Also, we wanted to check to what extent the cuticles of glandular cells with discontinuities would be a barrier to the antibodies. The technique used allowed for the localization of de-esterified pectic homogalacturonans in the outer walls of gland cells. The remaining antibodies (which detect esterified pectins, hemicelluloses, and arabinogalactan protein) marked only debris or secretion residues on the gland or epidermal surfaces. Positive labeling with LM19 and CCRC-M38 antibodies suggests the presence of pectic homogalacturonan in the very superficial part of the glands’ cell walls, so they were easily accessible to antibodies.

## 1. Introduction

Carnivorous *Nepenthes* L. (tropical pitcher plants) are specialized carnivorous plants, which produce modified leaves in the form of pitchers to attract and trap animals, preventing their escape from it, and later digestion of and nutrient absorption from prey bodies [1,2,3,4]. *Nepenthes* species are highly diverse regarding pitchers; thus, 12 types have been distinguished [5].

**Figure 1:**
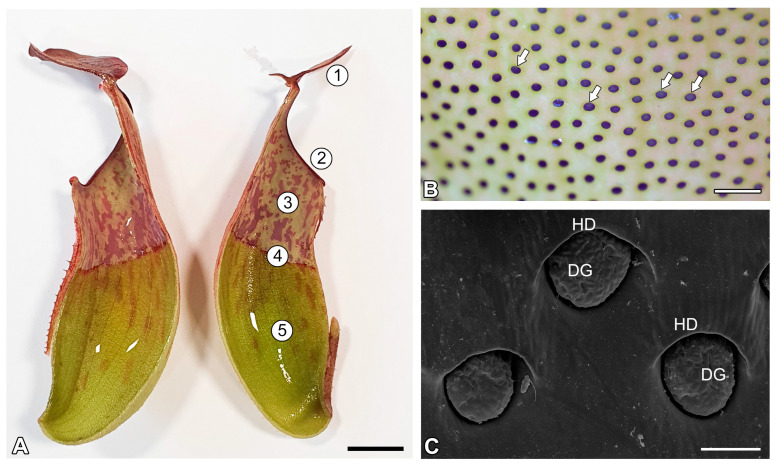
Pitcher zones and digestive glands, studied in *Nepenthes albomarginata* T.Lobb ex Lindl. (**A**) A dissected pitcher, with internal surfaces visible: 1. lid; 2. peristome; 3. slippery (wax) zone; 4. transitional zone; 5. digestive zone; bar = 1 cm. (**B**) Glands (arrows) from digestive zone, treated with toluidine blue; glands absorbed dye; bar = 1 mm. (**C**) Digestive gland morphology (scanning electron microscopy—SEM); digestive gland (DG); hood (HD); bar = 100 µm.

**Figure 2:**
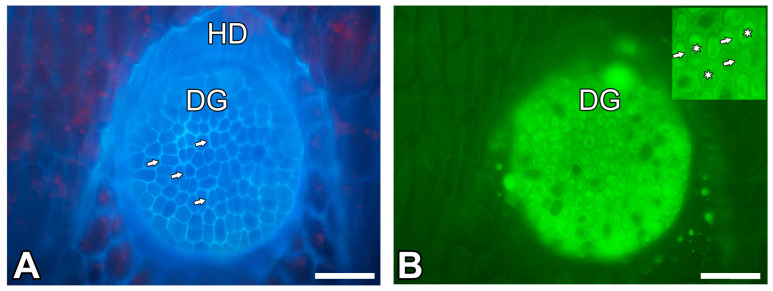
Structure of the digestive glands of the *Nepenthes albomarginata* pitcher. (**A**) A digestive gland, visible glandular cells, and strong blue autofluorescence of the radial cell walls (arrow) of these cells; bar = 50 µm. (**B**) The same gland as in A, stained with DiOC_6_; see green fluorescence of protoplasts (star); cell wall (arrow); bar = 50 µm.

The typical *Nepenthes* pitcher can be divided into structural and functional zones, which are related to attraction (lid and peristome with nectaries), retention (peristome, a wax zone), digestion and absorption (a zone with glands, on the bottom of the pitcher, Figure 1A–C). These zones differ in structure and have distinct macro- and micromorphological features (e.g., [1,6,7,8,9,10]). These zones may be strongly modified or completely reduced in some species (e.g., [6,11,12,13]); however, a digestion–absorption zone occurs in all examined species. The glands (Figure 1B,C and Figure 2A,B) from the digestion–absorption zone produce pitcher fluid which contains digestive enzymes (e.g., [14,15,16,17,18]). Enzyme presence was localized in digestive glands using cytochemical methods and substrate film technique [19,20,21,22]. However, according to Vassilyev and. Muravnik [23], also nectaries of the lid produce the digestive fluid into the pitcher in the growing still-closed pitchers of *Nepenthes khasiana* Hook.f. Vassilyev [24] proposed that the nectaries of the peristome in the closed pitchers of *Nepenthes khasiana* secrete polysacharide slime. So, at least in *Nepenthes khasiana*, the fluid in the pitcher is a mixture of secretions from different types of glands.

Juniper et al. [1] classified the digestive glands of *Nepenthes* as sessile glands, which lie on the epidermis. The digestive *Nepenthes* gland consists of several layers of glandular cells, a layer or layers of endodermal cells (see schematic Figure 18 in Owen et al. [25]), and basal cells that are in contact with vascular tissues [6,22,25,26].

Schnepf [27] described loose cuticles (made of cutin droplets) in the digestive glands of *Nepenthes*. According to Gorb et al. [7], in *Nepenthes* × *ventrata* Hort. ex Fleming digestive gland, there is a thin, up to 1 µm thick cuticle. Gorb and Gorb studied *Nepenthes alata* Blanco pitcher structure using cryo-SEM. They found that the upper cell layer of *Nepenthes alata* digestive glands bears extracellular coverage, which shows no evident layers, but has a coarse reticulate structure and consists of extremely inhomogeneous material [28]. According to Owen et al. [25], a thin cuticle layer occurs on the surface of this species’ outer cells of glands. These authors also observed that the outer gland cell wall was filled with cutinized deposits. Such a structure fits the cuticle model in *Nepenthes* glands proposed by Juniper et al. [1] based on the research of Russian researchers A.E. Vassilyev and Lyudmila E. Muravnik, who analyzed *Nepenthes khasiana*. Goebel has already shown that pitchers can take up organic compounds [6]. Still, it was Owen et al. [25] who showed experimentally how compounds are taken up by the symplastic and apoplastic pathways, respectively, using 6(5)carboxyfluorescein and lanthanum. Ivesic et al. [29] showed that the digestive glands of *Nepenthes* × *ventrata* may adsorb nutrients partly by endocytosis; thus, even whole protein molecules can be absorbed. Therefore, cuticle discontinuities of *Nepenthes* glands allow both secretion and absorption, and the cuticle of these glands is not a barrier. This study aimed to fill the gap in the literature concerning the immunocytochemistry of *Nepenthes* digestive glands in the major cell wall polysaccharides and glycoproteins. Cell wall components such as pectins (including pectic homogalacturonans), hemicelluloses, and arabinogalactans influence the structure of the wall and its properties. They also affect the ability to transport substances through the cell wall, which is particularly important for carnivorous plants, which must transport enzymes and then nutrients from their prey through the cell wall. However, the main aim was to check to what extent a cuticle with discontinuities would be a barrier to the antibodies in these *Nepenthes* digestive glands.

## 2. Results

### 2.1. Cuticle and Cuticular Discontinuities

Solutions of toluidine blue were used to test for permeable cuticles (occurrence of cuticular discontinuities). Digestive glands were intensively stained by toluidine blue, but epidermal cells in between did not take up the stain (Figure 1B).

**Figure 3:**
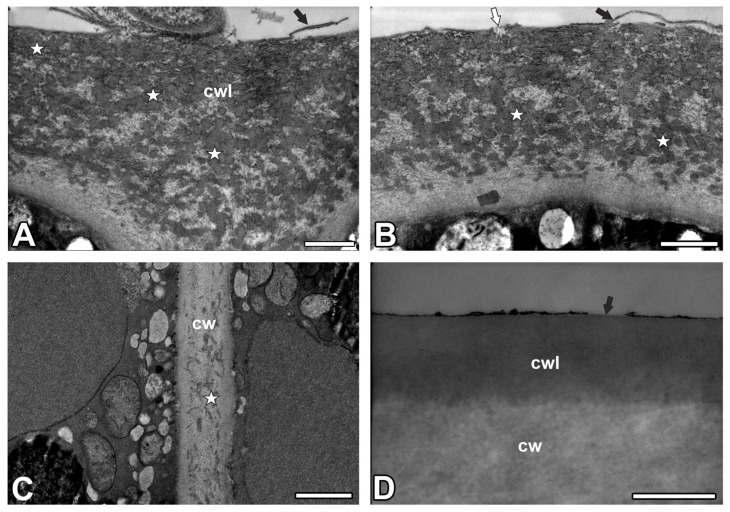
Cuticle and cuticular discontinuities of the digestive glands and epidermal cells of the *Nepenthes albomarginata*. (**A**,**B**) Outer cell wall and cuticle of glandular cell of digestive gland; cutinized wall layer (cwl); cutin cystoliths (star); cuticular gaps (white arrow); cuticularized layer (black arrow); bar = 500 nm. (**C**) Radial cell walls (cw) of a glandular cell of the digestive gland; cutin cystoliths (star); bar = 1 µm. (**D**) Outer cell wall and cuticle of epidermal cell from digestive zone of pitcher; note the thick cutinized wall layer (cwl); cuticularized layer (black arrow); cell wall (cw); bar = 250 µm.

The digestive gland had a thin cuticularized layer, which was ruptured in some places. The cutinized wall layer was very thick, with a very dense network of cutin cystoliths (cuticular droplets) (Figure 3A,B). Between cystoliths, there was non-impregnated wall material. The cuticular gaps occurred (Figure 3A,B). The innermost layer of the cell wall did not have cutin cystoliths. However, cutin cystoliths occurred in radial cell walls (Figure 3C). These cell walls had strong autofluorescence under UV (Figure 2A). In the epidermal cells, there were no cuticle discontinuities (Figure 3D).

### 2.2. Pectic Homogalacturonan Distribution

**Figure 4:**
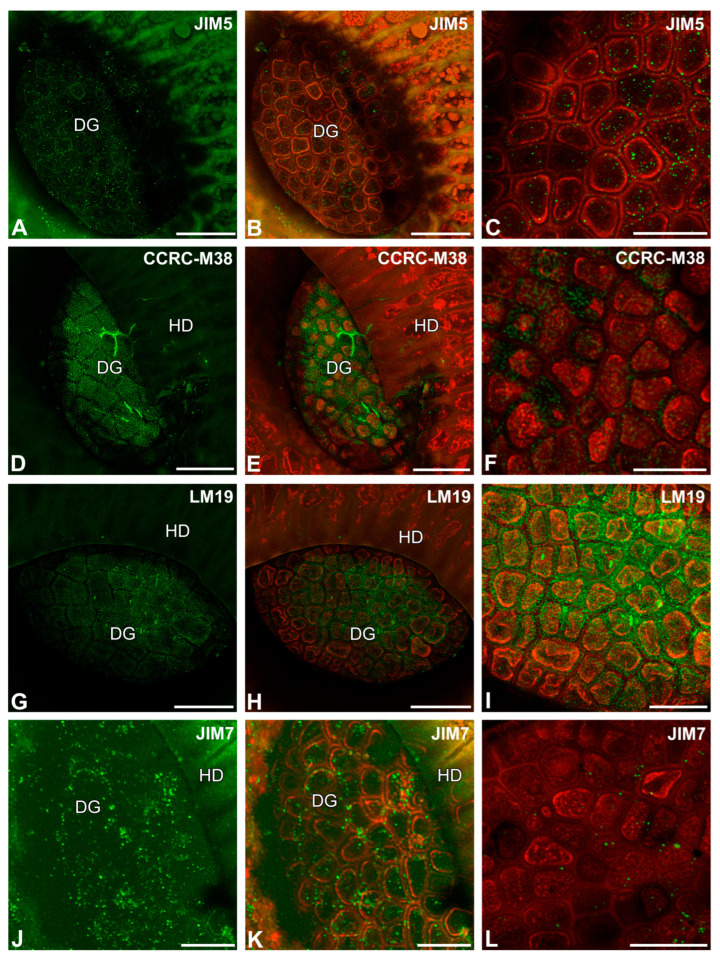
Pectic homogalacturonan detected in the digestive gland of the *Nepenthes albomarginata* pitcher; digestive gland (DG), hood (HD) (intense green color—signal of antibody; red-brown color—autofluorescence). (**A**,**B**) Labeling of cells with JIM5 (low methylesterified HG), bar = 50 µm. (**C**) Labeling of cells with JIM5 antibody, glandular cells of digestive gland, bar = 25 µm. (**D**,**E**) Labeling of cells with CCRC-M38 antibody (a fully de-esterified HGs), note the positive signal in the form of a network, bar = 50 µm. (**F**) Labeling of cells with CCRC-M38 antibody, glandular cells of digestive gland, note the positive signal in the form of a network, bar = 25 µm. (**G**,**H**) Labeling of cells with LM19 antibody (low methylesterified HG), note the positive signal in the form of a network, bar = 50 µm. (**I**) Labeling of cells with LM19 antibody, glandular cells of digestive gland, note the positive signal in the form of a network, bar = 25 µm. (**J**,**K**) Labeling of cells with JIM7 antibody (high methylesterified HG), bar = 25 µm. (**L**) Labeling of cells with JIM7 antibody, glandular cells of digestive gland, bar = 25 µm.

After immunolabeling with the JIM5 antibody (low methylesterified HGs), the signal was observed as dots on the surfaces of digestive gland cells and some epidermal cells (Figure 4A–C). The fluorescence signals detected by CCRC-M38 (a fully de-esterified HGs) (Figure 4D–F) and by LM19 (low methylesterified HG) were observed as a meshwork in the outer cell walls of digestive glands (Figure 4G–I). However, a fluorescence signal from highly esterified HGs (detected by JIM7) was observed as dots on the surfaces of digestive gland cells (Figure 4J–L).

### 2.3. Hemicellulose Distribution

**Figure 5:**
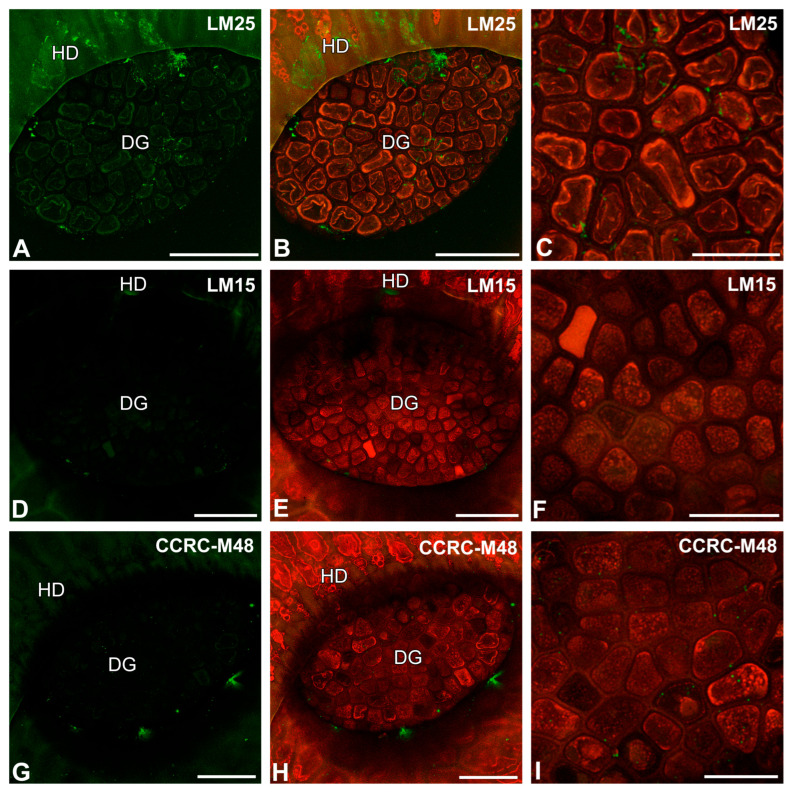
Hemicelluloses detected in the digestive gland of the *Nepenthes albomarginata* pitcher; digestive gland (DG), hood (HD) (intense green color—signal of antibody; red-brown color—autofluorescence). (**A**,**B**) Labeling of cells with LM25 antibody (xyloglu galactoxyloglucan), bar = 50 µm. (**C**) Labeling of cells with LM25 antibody, glandular cells of digestive gland, bar = 25 µm. (**D**,**E**) Labeling of cells with LM15 (xyloglucan), bar = 50 µm. (**F**) Labeling of cells with LM15 antibody, glandular cells of the digestive gland, bar = 25 µm. (**G**,**H**) Labeling of cells with CCRC-M48 antibody (xyloglucan), bar = 50 µm. (**I**) Labeling of cells with CCRC-M48 antibody glandular cells of the digestive gland, note the positive signal in the form of a network, bar = 25 µm.

**Figure 6:**
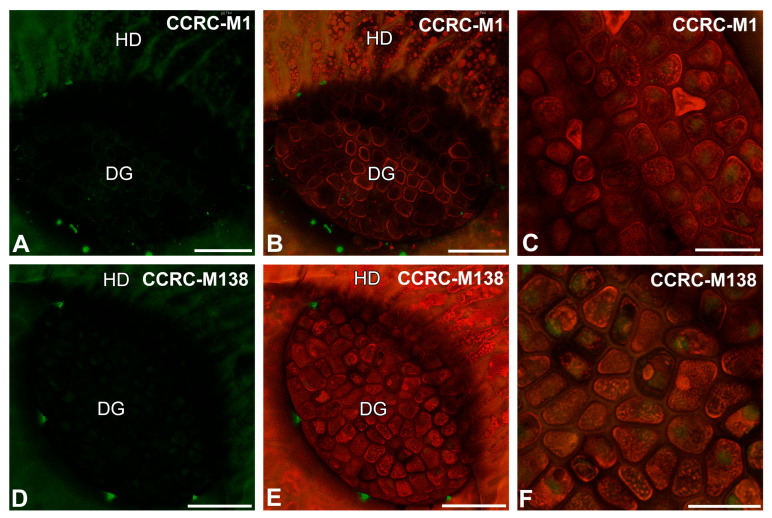
Hemicelluloses detected in the digestive gland of the *Nepenthes albomarginata* pitcher; digestive gland (DG), hood (HD) (intense green color—signal of antibody; red-brown color—autofluorescence). (**A**,**B**) Labeling of cells with CCRC-M1 antibody (xyloglucan), bar = 50 µm. (**C**) Labeling of cells with CCRC-M1 antibody, glandular cells of the digestive gland, bar = 25 µm. (**D**,**E**) Labeling of cells with CCRC-M138 antibody (xylan), note the positive signal in the form of a network, bar = 50 µm. (**F**) Labeling of cells with CCRC-M138 antibody, glandular cells of digestive gland, note the positive signal in the form of a network, bar = 25 µm.

After immunolabeling with the LM25 antibody (which recognizes land plants galactoxyloglucan), the signal of this antibody was observed in secretion or debris (debris is understood as non-deified remains of organisms and secretions that are in a trap) on the digestive gland and epidermal cell surfaces (Figure 5A–C). After immunolabeling with the LM15 antibody (which reacts with xyloglucan), the signal of this antibody was not observed in cell walls (Figure 5D–F). The LM15 antibody yielded fluorescence signals in the debris on the cell surfaces (Figure 5D,E). After immunolabeling with the CCRC-M48 antibody (which reacts with xyloglucan and recognizes the XXLG, XLLG glycan group of Non-Fucosylated Xyloglucan-5), the signal was not observed in cell walls (Figure 5G–I) but occurred in the debris on the cell surfaces (Figure 5G–I). After immunolabeling with the CCRC-M1 antibody (which reacts with xyloglucan and recognizes the alpha-Fuc-(1,2)-beta-Gal glycan group of Fucosylated Xyloglucan), the signal was not observed in cell walls (Figure 6A–C). The CCRC-M1 antibody yielded fluorescence signals in the debris on the cell surfaces (Figure 6A,B). After immunolabeling with the CCRC-M138 antibody (which recognizes the glycan group of Xylan-6), the signal was not observed in the cell walls (Figure 6D–F). The CCRC-M138 antibody yielded fluorescence signals in the debris on the cell surfaces (Figure 6D,E).

**Figure 7:**
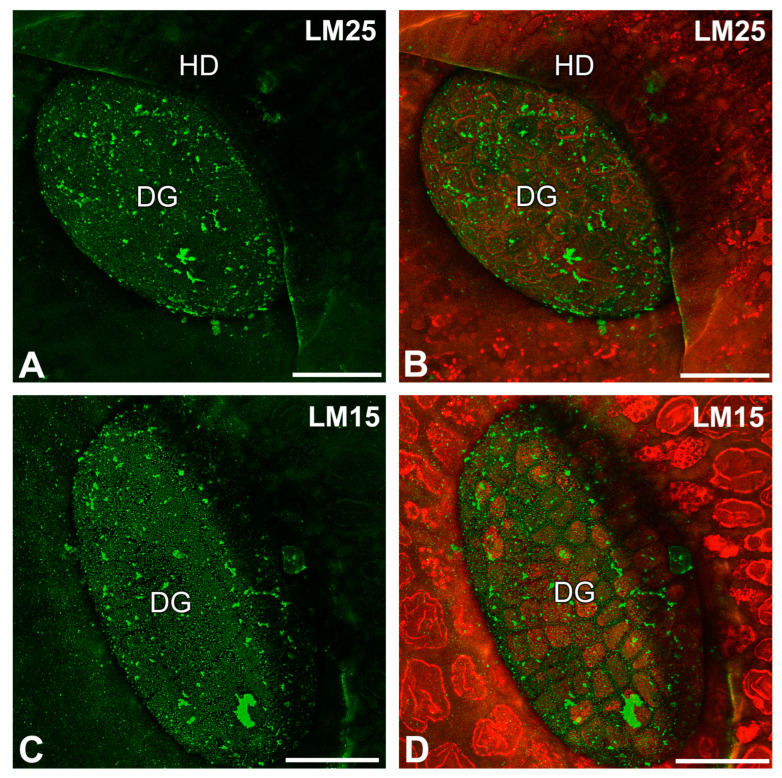
Hemicelluloses detected in the digestive gland of the *Nepenthes albomarginata* pitcher, after pre-treated with pectate lyase; digestive gland (DG), hood (HD) (intense green color—signal of antibody; red-brown color—autofluorescence). (**A**,**B**) Labeling of cells with LM25, bar = 50 µm. (**C**,**D**) Labeling of cells with LM15, bar = 50 µm.

When the sections were pre-treated with pectate lyase (during which pectins were removed), the fluorescence signals detected by LM25 and LM15 antibodies were observed as a meshwork in the outer cell walls of digestive glands and debris (Figure 7A–D). 

### 2.4. The Arabinogalactan Protein (AGP) Distribution

**Figure 8:**
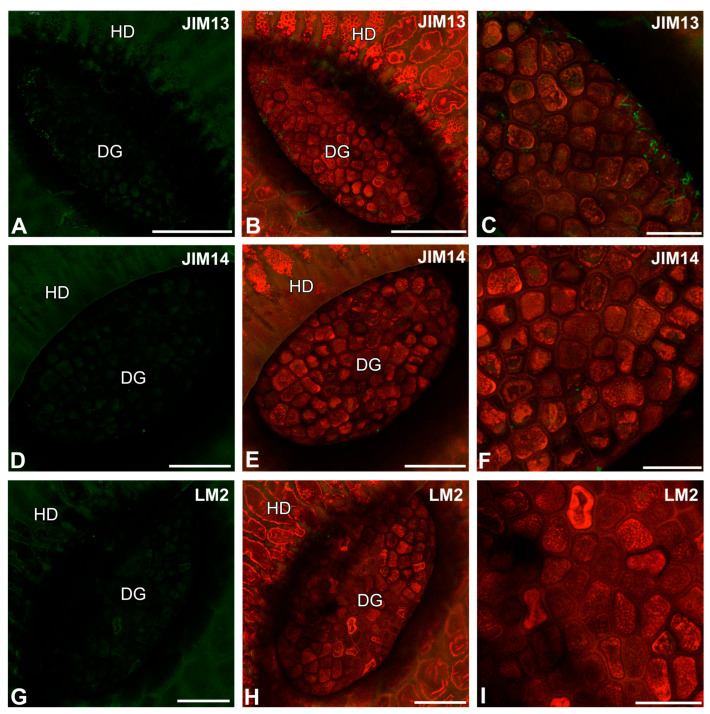
Arabinogalactan proteins detected in the digestive gland of the *Nepenthes albomarginata* pitcher; digestive gland (DG), hood (HD) (intense green color—signal of antibody; red-brown color—autofluorescence). (**A**,**B**) Labeling of cells with JIM13 antibody, bar = 50 µm. (**C**) Labeling of cells with JIM13 antibody, glandular cells of digestive gland, bar = 25 µm. (**D**,**E**) Labeling of cells with JIM14 antibody, note the positive signal in the form of a network, bar = 50 µm. (**F**) Labeling of cells with JIM14 antibody, glandular cells of digestive gland, bar = 25 µm. (**G**,**H**) Labeling of cells with LM2 antibody, bar = 50 µm. (**I**) Labeling of cells with LM2 antibody glandular cells of the digestive gland, bar = 25 µm.

After immunolabeling with the JIM13 antibody, the signal of this antibody was observed in debris or bacteria on the digestive gland cell surfaces (Figure 8A–C). The immunolabeling results with the JIM14 antibody (Figure 8D,E) and the LM2 antibody (Figure 8G–I) were similar. The signal was rare and observed as dots on the cell surfaces.

## 3. Discussion

### 3.1. Cuticle Structure

To survive in nutrient-poor habitats, carnivorous plants have developed an astonishingly diverse digestive system [1,30]. Carnivorous plants have also developed various modifications of the cuticle in their glands to secrete mucus and enzymes and absorb nutrients from their prey [1]. Cuticle discontinuities may be developed differently and take the form of cuticular pores [31,32,33,34,35], cuticular gaps [36,37,38], and ‘ill-defined discontinuities’, all of which allow rapid movement across the leaf surface. In some species, both cuticular gaps and cuticular pores may occur: *Roridula* [39] and *Utricularia* (Joel and Juniper [36] interpretation of results of Finerean and Lee [40]). The specific, giant cuticular discontinuities occur in *Byblis* glands, forming cuticular holes [41]. The presence of cuticle discontinuities can be checked using vital dyes, as was demonstrated in the digestive glands of *Nepenthes* × *coccinea*, *Nepenthes* × *ventrata* by Adlassnig et al. [42], and here in the digestive glands of *Nepenthes albomarginata*.

We found that the cuticle of the digestive glands in *Nepenthes albomarginata* is similar in structure (presence of cutin cystoliths) to the cuticle of the glands in *Nepenthes* spp. [27], *Nepenthes khasiana* [1], and *Nepenthes alata* [25]. According to Juniper et al. 1989 [1], the cuticularized layer undergoes exfoliation during gland maturation, but we still observed it in *Nepenthes albomarginata* glands.

Only a few *Nepenthes* species have been studied in detail regarding cuticle structure. *Nepenthes* species are an extremely diverse group with different strategies for obtaining nutrient compounds and with various specializations in the morphology and function of pitchers [5,11,43,44,45]; therefore, in the future, it should be investigated whether glands from such different types of pitchers have a similar cuticle structure. It will be interesting to examine the structure of cell walls in glandular structures in other species that have pitcher traps: *Sarracenia*, *Heliamphora*, *Darlingtonia*, and *Cephalotus* [1,3].

### 3.2. Whole-Mount Immunolabeled Gland Technique

Whole-mount immunolabeled organ technique was used successfully in the analysis of plant organs lacking cuticles, such as pollen tubes [46,47] and root hairs [48,49,50]. Li et al. [46] showed arabinogalactan epitopes in the cell wall of pollen tubes of *Nicotiana tabacum* L. Chebli et al. [47] showed pectin epitopes in the cell wall of pollen tubes of *Arabidopsis thaliana* L. Larson et al. [49] showed that even live *Arabidopsis thaliana* root hairs may be labeled with cell wall polymer-specific antibodies. Marzec et al. [50] detected the LM2 epitope on the surface of primordia and root hair tubes in plants able to generate root hairs in *Hordeum vulgare* L. using whole-mount immunolabelled root sections, which were chemically fixed. As for carnivorous plants, the whole-mount immunolabeled organ technique was used twice. In case of *Utricularia dichotoma* subsp. *novae-zelandiae* (Hook.f) R.W.Jobson, the antibodies penetrated the cell wall only in the areas where the cuticle had an open structure; however, no labeling occurred in cell wall parts which were heavily impregnated with cutin [51,52]. Płachno and Kapusta [51], using whole-mount immunolabeled traps, found that the cell walls of the quadrifid arms were especially rich in low-methyl-esterified homogalacturonan. Also, arabinogalactan proteins were detected. Hemicelluloses were detected when the traps were pre-treated with pectate lyase (during which pectins were removed). This was caused by the fact that pectic homogalacturonans mask abundant sets of xyloglucan epitopes in plant cell walls [53]. The next examined species, *Drosophyllum lusitanicum* (L.) Link, is interesting because it relates to *Nepenthes* [54,55]. Płachno et al. [38], using whole-mount immunolabeled glands, studied the occurrence of wall components in this species. Despite the presence of cuticle gaps, antibody penetration was limited only to the cell wall surface (e.g., AGPs labeled with JIM8, pectic homogalacturonan labeled with LM19, galactoxyloglucan labeled with LM25, and xyloglucan labeled with LM15). It should be noted that some results were ambiguous, and it was sometimes difficult to distinguish whether the signal came from the peripheral part of the wall or from the secretion or the material on the cell surface.

Like *Utricularia dichotoma* and *Drosophyllum lusitanicum* glands, we found pectic low-methyl-esterified homogalacturonans in the outer cell walls of *Nepenthes albomarginata*. Positive labeling with LM19 and CCRC-M38 antibodies suggests the presence of pectic homogalacturonan in the very superficial part of the glands’ cell walls, so they were easily accessible to antibodies. Pectic homogalacturonans are known to be involved in plant cell wall porosity and hydration [56,57]. We suggest that the presence of these hydrophilic homogalacturonans on the gland surface may facilitate the processes of fluid secretion into the pitcher or absorption of compounds produced due to the digestion of prey. Recently pectic homogalacturonans and hemicelluloses were detected in cell walls of cells in *Nepenthes* external glands, which may function as may act as hydathodes or hydropotes [58].

We treat the technique used as a preliminary research stage on *Nepenthes* glands. Because the antibodies, which detect esterified pectins, hemicelluloses, and arabinogalactan protein marked only debris or secretion residues on the gland or epidermal surfaces, which may result either from a lack of epitopes in external parts of cell walls of glands or difficulties with antibody penetration. In the next stage, we plan to use cross-sections through the glands and also enzyme treatment, which will allow better access of antibodies to epitopes and provide a comprehensive picture of the occurrence of the major cell wall polysaccharides and glycoproteins.

## 4. Materials and Methods

### 4.1. Plant Material

*Nepenthes albomarginata* T.Lobb ex Lindl. pitchers were taken from the first author’s collection; they were grown in the humid terrarium.

### 4.2. Immunochemical Analysis

The bottom parts of pitchers (digestive zone) were cut for small fragments and fixed in 8% (*w*/*v*) formaldehyde (Sigma-Aldrich, Sigma-Aldrich Sp. zo.o. Poznań, Poland) mixed with 0.25% (*v*/*v*) glutaraldehyde (Sigma-Aldrich, Sigma-Aldrich Sp. zo.o. Poznań, Poland) in a PIPES buffer with addition of Tween (Sigma-Aldrich, Sigma-Aldrich Sp. zo.o. Poznań, Poland) or DMSO (Sigma-Aldrich, Sigma-Aldrich Sp. zo.o. Poznań, Poland).

The plant material was washed in a PBS buffer and later blocked with 1% bovine serum albumin (BSA, Sigma-Aldrich) in a PBS buffer and incubated with the following primary antibodies overnight at 4 °C: anti-homogalacturonans (HGs): JIM5 (low methylesterified HG), JIM7 (highly esterified HG), LM19 (low methylesterified HG), and CCRC-M38 (a fully de-esterified HG); and anti-hemicelluloses: LM25 (galactoxyloglucan), CCRC-M48 (xyloglucan), CCRC-M1 (xyloglucan), LM15 (xyloglucan), and CCRC-M138 (xylan) [59,60,61,62,63,64], Paul Knox, PhD, University of Leeds. Available online: https://www.kerafast.com/cat/799/paul-knox-phd (accessed on 13 November 2023), https://www.kerafast.com/(accessed on 13 November 2023), anti-arabinogalactan protein: JIM13, JIM14, and LM2 [65]. All of the primary antibodies were used in a 1:20 dilution. They were purchased from Plant Probes, UK (rat monoclonal antibodies: JIM5, JIM7, LM19, LM25, LM15, JIM13, JIM14, and LM2) and Agrisera, Sweden (mouse monoclonal antibodies: CCRC-M38, CCRC-M1, CCRC-M48, and CCRC-M138). Secondary antibodies: goat anti-rat secondary or anti-mouse antibody conjugated with FITC or Alexa Fluor 488, respectively, were purchased from Abcam (Cambridge, UK). The samples were then cover-slipped using a Mowiol mounting medium: a mixture of Mowiol ^®^4-88 (Sigma-Aldrich) and glycerol for fluorescence microscopy (Merck, Warsaw, Poland) with the addition of 2.5% DABCO (Carl Roth GmbH + Co. KG, Karlsruhe, Germany). They were viewed using a Leica STELLARIS 5 WLL confocal microscope with Lightning module deconvolution. Negative controls were created by omitting the primary antibody step, which caused no fluorescence signal in any of the control frames for any stained slides (Figure S1). To remove the HG from the cell walls, the material was pretreated with 0.1 M sodium carbonate pH = 11.4 for 2 h at room temperature. This was followed by digestion with a pectate lyase 1A (Nzytech) at 10 μg/mL for 2 h at room temperature in 50 mM N-cyclohexyl-3-aminopropane sulfonic acid (CAPS) with the addition of 2 mM of a CaCl_2_ buffer at pH 10, and then incubation with the antibodies, as described above.

### 4.3. Scanning Transmission Electron Microscopy

The glands were also examined using electron microscopy, as follows: Fragments of the traps were fixed in a mixture of 2.5% glutaraldehyde with 2.5% formaldehyde in a 0.05 M cacodylate buffer (Sigma-Aldrich, Sigma-Aldrich Sp. z o.o., Poznań, Poland; pH 7.2) for a few days, and later, the material was processed as in Płachno et al. [66]. The material was dehydrated with acetone and embedded in an Epoxy Embedding Medium Kit (Fluka) or Durcupan resin (Sigma-Aldrich Chemie GmbH, Taufkirchen, Germany). Ultrathin sections were cut on a Leica Ultracut UCT ultramicrotome. The sections were examined using a Hitachi UHR FE-SEM SU 8010 microscope housed at the University of Silesia in Katowice.

### 4.4. Scanning Electron Microscopy

For the scanning electron microscopy (SEM), the traps were cut and fixed in methanol, later transferred to ethanol, then transferred to acetone, and dried using supercritical CO_2_. The material was then sputter-coated with gold and examined using a Hitachi UHR FE-SEM SU 8010 microscope housed at the University of Silesia in Katowice.

### 4.5. Light Microscopy (LM)

The living glands were stained with 20 μM DiOC_6_ (3,3′-dihexyloxacarbocyanine iodide; Thermo Fisher, Rockland, MD, USA) dissolved in water [67]. The traps were examined using a Nikon Eclipse E400 light microscope. Autofluorescence of gland cell walls was examined using a Nikon Eclipse E400 light microscope (Tokyo, Japan) with a UV-2A filter (Ex. 330–380 nm, DM. 400 nm, Em. 420-α nm). A toluidine blue solution was applied to the pitcher to see if glands can take up aqueous solutions. After several hours, the pitcher was rinsed with water, and photos were taken using Emspira 3 Digital Microscope (Leica, PIK Instruments Sp. z o.o. Piaseczno, Poland).

## 5. Conclusions

The method used was not perfect (because not all antibodies gave positive results, which may result from a lack of epitopes or difficulties with antibody penetration). Still, it did show the presence of pectic homogalacturonans in the very superficial part of the cell walls of the glands. Our results contribute to the current knowledge regarding cell wall structure and cuticle structure in carnivorous plant glands.

## Data Availability

The data presented in this study are available on request from the corresponding author.

## Supplementary Materials

The following supporting information can be downloaded at: https://www.mdpi.com/article/10.3390/ijms26189174/s1.

## References

[1] Juniper B.E., Robbins R.J., Joel D.M. (1989). The Carnivorous Plants.

[2] Pavlovič A., Masarovičová E., Hudák J. (2007). Carnivorous syndrome in Asian pitcher plants of the genus *Nepenthes*. Ann. Bot..

[3] Król E., Płachno B.J., Adamec L., Stolarz M., Dziubińska H., Trębacz K. (2012). Quite a few reasons for calling carnivores ‘the most wonderful plants in the world’. Ann. Bot..

[4] Moran J.A., Clarke C.M. (2010). The carnivorous syndrome in *Nepenthes* pitcher plants: Current state of knowledge and potential future directions. Plant Signal. Behav..

[5] Cheek M., Jebb M., Murphy B. (2019). A classification of functional pitcher types in *Nepenthes* (Nepenthaceae). bioRxiv.

[6] Lloyd F.E. (1942). The Carnivorous Plants.

[7] Gorb E., Kastner V., Peressadko A., Arzt E., Gaume L., Rowe N., Gorb S. (2004). Structure and properties of the glandular surface in the digestive zone of the pitcher in the carnivorous plant *Nepenthes ventrata* and its role in insect trapping and retention. J. Exp. Biol..

[8] Gorb E.V., Gorb S.N. (2006). Physicochemical properties of functional surfaces in pitchers of the carnivorous plant *Nepenthes alata* Blanco (Nepenthaceae). Plant Biol..

[9] Bauer U., Jetter R., Poppinga S., Ellison A.M., Adamec L. (2018). Non-motile traps. Carnivorous Plants: Physiology, Ecology, and Evolution.

[10] Lessware O.C., Mantell J.M., Bauer U. (2025). Carnivorous *Nepenthes* pitcher plants combine common developmental processes to make a complex epidermal trapping surface. Ann. Bot..

[11] Bauer U., Clemente C.J., Renner T., Federle W. (2012). Form follows function: Morphological diversification and alternative trapping strategies in carnivorous *Nepenthes* pitcher plants. J. Evol. Biol..

[12] Lenz A.K., Bauer U. (2022). Pitcher geometry facilitates extrinsically powered ‘springboard trapping’ in carnivorous *Nepenthes gracilis* pitcher plants. Biol. Lett..

[13] Chomicki G., Burin G., Busta L., Gozdzik J., Jetter R., Mortimer B., Bauer U. (2024). Convergence in carnivorous pitcher plants reveals a mechanism for composite trait evolution. Science.

[14] Amagase S. (1972). Digestive enzymes in insectivorous plants: III. Acid proteases in the genus *Nepenthes* and *Drosera peltata*. J. Bioch..

[15] Tökés Z.A., Woon W.C., Chambers S.M. (1974). Digestive enzymes secreted by the carnivorous plant *Nepenthes macferlanei* L.. Planta.

[16] Hatano N., Hamada T. (2008). Proteome analysis of pitcher fluid of the carnivorous plant *Nepenthes alata*. J. Prot. Res..

[17] Buch F., Kaman W.E., Bikker F.J., Yilamujiang A., Mithöfer A. (2015). Nepenthesin protease activity indicates digestive fluid dynamics in carnivorous *Nepenthes* plants. PLoS ONE.

[18] Saganová M., Bokor B., Stolárik T., Pavlovič A. (2018). Regulation of enzyme activities in carnivorous pitcher plants of the genus *Nepenthes*. Planta.

[19] Heslop-Harrison Y., Dingle J.T., Dean R.T. (1975). Enzyme release in carnivorous plants. Lysozymes in Biology and Pathology.

[20] Parkes D.M. (1980). Adaptive Mechanisms of Surface and Glands in Some Carnivorous Plants. Master’s Thesis.

[21] Płachno B.J., Adamec L., Lichtscheidl I.K., Peroutka M., Adlassnig W., Vrba J. (2006). Fluorescence labelling of phosphatase activity in digestive glands of carnivorous plants. Plant Biol..

[22] Thornhill A.H., Harper I.S., Hallam N.D. (2008). The development of the digestive glands and enzymes in the pitchers of three Nepenthes species: *N. alata*, *N. tobaica*, and *N. ventricosa* (Nepenthaceae). Int. J. Plant. Sci..

[23] Vassilyev A.E., Muravnik L.E. (2007). The nectaries of the lid in closed pitchers of *Nepenthes khasiana* (Nepenthaceae) secrete a digestive fluid. Bot. Zhurnal.

[24] Vassilyev A.E. (2007). The nectaries of the peristome in the closed pitchers of *Nepenthes khasiana* (Nepenthaceae) secrete polysacharide slime. Bot. Zhurnal.

[25] Owen T.P., Lennon K.A., Santo M.J., Anderson A.Y. (1999). Pathways for nutrient transport in the pitchers of the carnivorous plant *Nepenthes alata*. Ann. Bot..

[26] Fenner C.A. (1904). Beitraege zur Kenntnis der Anatomie, Entwicklungsgeschichte und Biologie der Laubblaetter und Druesen einiger Insektivoren. Flora.

[27] Schnepf E. (1965). Die Morphologie der Sekretion in pflanzlichen Drüsen. Ber. Dtsch. Bot. Ges..

[28] Gorb E.V., Gorb S.N., Gorb S.N. (2009). Functional Surfaces in the Pitcher of the Carnivorous Plant *Nepenthes alata*: A Cryo-Sem Approach. Functional Surfaces in Biology.

[29] Ivesic C., Krammer S., Koller-Peroutka M., Laarouchi A., Gruber D., Lang I., Lichtscheidl I.K., Adlassnig W. (2023). Quantification of Protein Uptake by Endocytosis in Carnivorous Nepenthales. Plants.

[30] Freund M., Graus D., Fleischmann A., Gilbert K.J., Lin Q., Renner T., Stigloher C., Albert V.A., Hedrich R., Fukushima K. (2022). The digestive systems of carnivorous plants. Plant Physiol..

[31] Williams S.E., Pickard B.G. (1969). Secretion, absorption and cuticular structure in *Drosera* tentacles. Plant Physiol..

[32] Ragetli H.W., Weintraub L.O.E. (1972). Characteristics of *Drosera* tentacles. I. Anatomical and cytological details. Can. J. Bot..

[33] Williams S.E., Pickard B.G. (1974). Connections and barriers between cells of *Drosera* tentacles in relation to their electrophysiology. Planta.

[34] Płachno B.J., Faber J., Jankun A. (2005). Cuticular discontinuities in glandular hairs of *Genlisea* St.-Hil. in relation to their functions. Acta Bot. Gall..

[35] Płachno B.J., Kozieradzka-Kiszkurno M., Świątek P. (2007). Functional Ultrastructure of *Genlisea* (Lentibulariaceae) Digestive Hairs. Ann. Bot..

[36] Joel D.M., Juniper B.E., Cutler D.F., Alvin K.L., Price C.E. (1982). Cuticular gaps in carnivorous plant glands. The Plant Cuticle.

[37] Joel D.M., Rea P.A., Juniper B.E. (1983). The cuticle of *Dionaea muscipula* Ellis (Venus’s Flytrap) in relation to stimulation, secretion and absorption. Protoplasma.

[38] Płachno B.J., Kapusta M., Stolarczyk P., Świątek P. (2024). Do Cuticular Gaps Make It Possible to Study the Composition of the Cell Walls in the Glands of *Drosophyllum lusitanicum*?. Int. J. Mol. Sci..

[39] Anderson B. (2005). Adaptations to foliar absorption of faeces: A pathway in plant carnivory. Ann. Bot..

[40] Fineran B.A., Lee M.S.L. (1975). Organization of quadrifid and bifid hairs in the trap of *Utricularia monanthos*. Protoplasma.

[41] Płachno B.J., Lancelle S., Świątek P., Hepler P.K., Weidinger M., Lichtscheidl I. (2025). Cyto-architecture of *Byblis* glands and leaf cells based on freeze-substitution and conventional TEM. Ann. Bot..

[42] Adlassnig W., Koller-Peroutka M., Bauer S., Koshkin E., Lendl T., Lichtscheidl I.K. (2012). Endocytotic uptake of nutrients in carnivorous plants. Plant J..

[43] Bauer U., Grafe T.U., Federle W. (2011). Evidence for alternative trapping strategies in two forms of the pitcher plant, *Nepenthes rafflesiana*. J. Exp. Bot..

[44] Bauer U., Di Giusto B., Skepper J., Grafe T.U., Federle W. (2012). With a flick of the lid: A novel trapping mechanism in *Nepenthes gracilis* pitcher plants. PLoS ONE.

[45] McPherson S. (2023). Nepenthes: The Tropical Pitcher Plants.

[46] Li Y.Q., Bruun L., Pierson E.S., Cresti M. (1992). Periodic deposition of arabinogalactan epitopes in the cell wall of pollen tubes of *Nicotiana tabacum* L.. Planta.

[47] Chebli Y., Kaneda M., Zerzour R., Geitmann A. (2012). The cell wall of the *Arabidopsis* pollen tube--spatial distribution, recycling, and network formation of polysaccharides. Plant Physiol..

[48] Willats W.G., McCartney L., Knox J.P. (2001). In-situ analysis of pectic polysaccharides in seed mucilage and at the root surface of *Arabidopsis thaliana*. Planta.

[49] Larson E.R., Tierney M.L., Tinaz B., Domozych D.S. (2014). Using monoclonal antibodies to label living root hairs: A novel tool for studying cell wall microarchitecture and dynamics in *Arabidopsis*. Plant Methods.

[50] Marzec M., Szarejko I., Melzer M. (2015). Arabinogalactan proteins are involved in root hair development in barley. J. Exp. Bot..

[51] Płachno B.J., Kapusta M. (2024). The Localization of Cell Wall Components in the Quadrifids of Whole-Mount Immunolabeled *Utricularia dichotoma* Traps. Int. J. Mol. Sci..

[52] Płachno B.J., Kapusta M., Stolarczyk P., Feldo M., Świątek P. (2024). Do Arabinogalactan Proteins Occur in the Transfer Cells of *Utricularia dichotoma*?. Int. J. Mol. Sci..

[53] Marcus S.E., Verhertbruggen Y., Hervé C., Ordaz-Ortiz J.J., Farkas V., Pedersen H.L., Willats W.G., Knox J.P. (2008). Pectic homogalacturonan masks abundant sets of xyloglucan epitopes in plant cell walls. BMC Plant Biol..

[54] Heubl G., Bringmann G., Meimberg H. (2006). Molecular phylogeny and character evolution of carnivorous plant families in Caryophyllales—Revisited. Plant Biol..

[55] Walker J.F., Yang Y., Moore M.J., Mikenas J., Timoneda A., Brockington S.F., Smith S.A. (2017). Widespread paleopolyploidy, gene tree conflict, and recalcitrant relationships among the carnivorous Caryophyllales. Am. J. Bot..

[56] Ridley M.A., O’Neill D., Mohnen D. (2001). Pectins: Structure, biosynthesis, and oligogalac-turonide-related signaling. Phytochemistry.

[57] Peaucelle A., Braybrook S., Höfte H. (2012). Cell wall mechanics and growth control in plants: The role of pectins revisited. Front. Plant Sci..

[58] Płachno B.J., Kapusta M., Feldo M., Stolarczyk P., Małota K., Banaś K. (2025). External Glands of *Nepenthes* Traps: Structure and Potential Function. Int. J. Mol. Sci..

[59] Liners F., Letesson J.J., Didembourg C., Van Cutsem P. (1989). Monoclonal Antibodies against Pectin: Recognition of a Conformation Induced by Calcium. Plant Physiol..

[60] Paul Knox, PhD, University of Leeds. https://www.kerafast.com/cat/799/paul-knox-phd.

[61] Knox J.P., Day S., Roberts K. (1989). A set of cell surface glycoproteins forms an early marker of cell position, but not cell type, in the root apical meristem of *Daucus carota* L.. Development.

[62] Verhertbruggen Y., Marcus S.E., Haeger A., Ordaz-Ortiz J.J., Knox J.P. (2009). An extended set of monoclonal antibodies to pectic homogalacturonan. Carbohydr. Res..

[63] Pattathil S., Avci U., Baldwin D., Swennes A.G., McGill J.A., Popper Z., Bootten T., Albert A., Davis R.H., Chennareddy C. (2010). A comprehensive toolkit of plant cell wall gly-can-directed monoclonal antibodies. Plant Physiol..

[64] McCartney L., Marcus S.E., Knox J.P. (2005). Monoclonal antibodies to plant cell wall xylans and arabinoxylans. J. Histochem. Cytochem..

[65] https://www.kerafast.com/.

[66] Płachno B.J., Świątek P., Jobson R.W., Małota K., Brutkowski W. (2017). Serial block face SEM visualization of unusual plant nuclear tubular extensions in a carnivorous plant (*Utricularia*, Lentibulariaceae). Ann. Bot..

[67] Lichtscheidl I., Lancelle S., Weidinger M., Adlassnig W., Koller-Peroutka M., Bauer S., Krammer S., Hepler P.K. (2021). Gland cell responses to feeding in *Drosera capensis*, a carnivorous plant. Protoplasma.

