# Supplementary material for: The Localization of Cell Wall Components in the Whole-Mount Immunolabeled Nepenthes Digestive Glands
Source: Int J Mol Sci. 2025 Sep 19;26(18):9174. doi: 10.3390/ijms26189174 (PMC12470327; doi:10.3390/ijms26189174)

Figure S1

Figure S1. Control reactions of cell wall components after immunolabeling (green color – signal of antibody), (**A**-**B)**. Digestive gland: (**A**) An autofluorescence signal collected for the FITC channel. (**B**) An autofluorescence signal collected for the Alexa 488, both bars = 50 um.

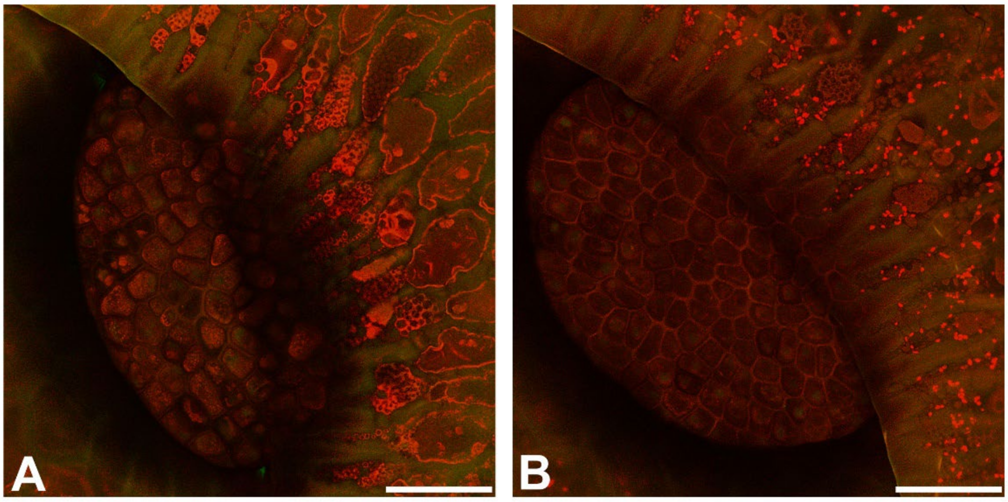

Supplement: Supplementary file 1 [file ijms-26-09174-s001.zip › ijms-3865866-supplementary.pdf]
